# Oral Treatment with γ-Aminobutyric Acid Improves Glucose Tolerance and Insulin Sensitivity by Inhibiting Inflammation in High Fat Diet-Fed Mice

**DOI:** 10.1371/journal.pone.0025338

**Published:** 2011-09-22

**Authors:** Jide Tian, Hoa N. Dang, Jing Yong, Wing-Sheung Chui, Matthew P. G. Dizon, Catherine K. Y. Yaw, Daniel L. Kaufman

**Affiliations:** Department of Molecular and Medical Pharmacology, University of California Los Angeles, Los Angeles, California, United States of America; Centre de Recherche Public de la Santé (CRP-Santé), Luxembourg

## Abstract

Adipocyte and β-cell dysfunction and macrophage-related chronic inflammation are critical for the development of obesity-related insulin resistance and type 2 diabetes mellitus (T2DM), which can be negatively regulated by Tregs. Our previous studies and those of others have shown that activation of γ-aminobutyric acid **(**GABA) receptors inhibits inflammation in mice. However, whether GABA could modulate high fat diet (HFD)-induced obesity, glucose intolerance and insulin resistance has not been explored. Here, we show that although oral treatment with GABA does not affect water and food consumption it inhibits the HFD-induced gain in body weights in C57BL/6 mice. Furthermore, oral treatment with GABA significantly reduced the concentrations of fasting blood glucose, and improved glucose tolerance and insulin sensitivity in the HFD-fed mice. More importantly, after the onset of obesity and T2DM, oral treatment with GABA inhibited the continual HFD-induced gain in body weights, reduced the concentrations of fasting blood glucose and improved glucose tolerance and insulin sensitivity in mice. In addition, oral treatment with GABA reduced the epididymal fat mass, adipocyte size, and the frequency of macrophage infiltrates in the adipose tissues of HFD-fed mice. Notably, oral treatment with GABA significantly increased the frequency of CD4^+^Foxp3^+^ Tregs in mice. Collectively, our data indicated that activation of peripheral GABA receptors inhibited the HFD-induced glucose intolerance, insulin resistance, and obesity by inhibiting obesity-related inflammation and up-regulating Treg responses *in vivo*. Given that GABA is safe for human consumption, activators of GABA receptors may be valuable for the prevention of obesity and intervention of T2DM in the clinic.

## Introduction

Obesity is associated with the development of type 2 diabetes mellitus (T2DM) and stems from the imbalance between calorie intake and expenditure, as well as genetic factors, leading to the accumulation of excess fat in the body. T2DM is characterized by impaired glucose tolerance, insulin resistance and insufficient insulin production by the pancreatic islet β-cells [1], [2]. The incidence of obesity-related T2DM is dramatically increasing worldwide. Individuals with obesity and T2DM are at risk of developing micro- and macrovascular diseases, such as hypertension, cardiovascular diseases and cerebovascular diseases [3], [4]. Although many medications are available for the management of hyperlipidemia and hyperglycemia, they fail to completely restore glucose homeostasis and/or have adverse effects. Therefore, the discovery and development of new reagents that can safely inhibit obesity development and improve glucose metabolism will be of great benefit for slowing the development of T2DM and limiting its long-term complications.

Previous studies have shown that adipocyte and β-cell dysfunction along with low-grade macrophage-related chronic inflammation are critical for the development of obesity-related insulin resistance and T2DM [2], [5], [6], [7], [8]. During the development of obesity and T2DM, adipocytes can produce adipokines and other factors, which recruit the infiltration of macrophages and other immunocompetent cells into the adipose tissues and affect insulin sensitivity in other organs, leading to low grade inflammation [9], [10], [11]. Apparently, inhibition of chronic inflammation and macrophage infiltration may inhibit adipocyte hypertrophy and improve glucose tolerance and insulin sensitivity. Indeed, regulatory T cells (Tregs) have been shown to inhibit the high fat diet (HFD)-induced adipocyte dysfunction, glucose intolerance, and insulin resistance in mice [12]. Notably, GABA_A_ receptors (GABA_A_-R) are expressed by adipose tissues and immunocompetent cells, such as macrophages and T cells [13], [14], [15]. Our previous studies and those of others have shown that activation of GABA_A_-R inhibits inflammatory diseases, such as type 1 diabetes (T1D), experimental autoimmune encephalomyelitis (EAE), collagen-induced rheumatoid arthritis (RA) in mice and prolongs the survival of syngenic islet grafts in diabetic NOD mice [16], [17], [18], [19], [20]. A recent study as well as our unpublished observations show that GABA promotes regeneration of the pancreatic β-cells and reverses hyperglycemia in the mouse model of T1D [20]. Accordingly, it is possible that activation of GABA receptors may modulate the HFD-induced adipocyte dysfunction and inflammation, as well as associated obesity and glucose metabolic disorder.

To test this hypothesis, we employed the HFD-induced obesity and T2DM model and treated orally with GABA to test whether activation of GABA receptors could prevent the HFD-induced obesity and T2DM, and improve glucose tolerance and insulin sensitivity after the onset of T2DM. We found that oral administration of GABA did not affect the amount of water and food consumption by animals, but reduced the HFD-induced gain in body weight and epididymal fat mass, accompanied by reducing the numbers of infiltrated macrophages in adipose tissues. Furthermore, treatment with GABA improved glucose tolerance and insulin sensitivity in mice, even after the onset of obesity and T2DM. Finally, we observed that treatment with GABA significantly increased the frequency of peripheral Tregs in mice, which are known to negatively regulate inflammation.

## Materials and Methods

### Mice and treatments

All experiments were approved by the Animal Research Committee of University of California, Los Angeles (protocol number 1993-211). Male C57BL/6 mice at 4 weeks of age were from Jackson Laboratories (Bar Harbor, ME, USA) and were housed in a specific pathogen free facility. The mice were fed with HFD beginning at 5 weeks of age (60% fat of caloric intake at 5.32 kcal/g, Research Diets, New Brunswick, USA) and provided with plain water (control) or water containing 2 mg/ml of GABA (Sigma, St. Louis, USA). The water bottles were changed weekly with fresh material. Their food intake and water consumption were measured weekly and their body weights, fasting blood glucose, glucose tolerance, and insulin sensitivity were measured longitudinally.

In addition, we tested whether treatment with GABA could modulate glucose intolerance and insulin resistance in mice after the onset of obesity and T2DM. C57BL/6 mice were fed with HFD and plain water for 20 weeks and tested for fasting blood glucose, glucose tolerance, and insulin sensitivity. Individual mice with obesity (body weights > 48 g) and T2DM (fasting blood glucose >145 mg/dL; blood glucose at 2 hours post-intraperitoneal glucose challenge >220 mg/dL; blood glucose at 30 min post insulin injection < 28% before insulin injection) were randomly divided and fed with HFD and plain water or water containing 2 mg of GABA for another 12 weeks, respectively. Their food intake and water consumption were measured weekly and their body weights, fasting blood glucose, glucose tolerance, and insulin sensitivity were measured longitudinally.

### Measurement of fasting blood glucose, glucose tolerance, and insulin sensitivity

The mice were fasted for 16 hours and the concentrations of blood glucose in individual mice were measured using an OneTouch ultra blood glucose meter (LifeScan, Milpitas, USA). Glucose tolerance in individual mice was injected intraperitoneally with 2 g/kg glucose (Sigma) and their blood glucose concentrations were measured at 15, 30, 60, 90, 120 and 180 min post challenge and the areas of under the curve (AUC) of blood glucose levels were calculated. For insulin tolerance tests, non-fasted individual mice were measured for the concentrations of blood glucose as 0 time point and injected intraperitoneally with 0.75 units/kg of human regular insulin (Eli Lilly, Indianapolis, USA). Subsequently, the concentrations of blood glucose were measured at 15, 30, 60, 90, 120 and 150 min post insulin injection. The changes in the percentage of blood glucose relative to the values at 0 time point for individual mice were calculated.

### Histological analysis of visceral adipose tissue

At the end of the experiments, mice were sacrificed and their visceral adipose tissue (VAT) were dissected and weighed. One portion of the VAT was fixed with 4% paraformaldehyde for 24 hours and the VAT sections at 5 µm were stained with Toluidine blue O. A total 5 sections from individual mice of each group (n = 8) were examined in a blinded fashion using the Lplab image analysis software.

Another portion of the VAT was fixed with the Bouin buffer for 48 hours and then paraffin-embedded. Their sections at 5 µm of 100 µm intervals were rehydrated, treated with 3% of H_2_0_2_ in methanol, and subjected to antigen retrieval with 0.01 M sodium citrate buffer (pH 6.0) in a high pressure-steamer. Subsequently, these sections were blocked sequentially with a biotin blocking system (Dako) and 20% of FBS in PBS. The sections were probed with biotinylated anti-F4/80 (PharMigen, San Diego, USA) overnight at 4°C. After washing, the immunocomplex was visualized using HRP-striptoavidin (Dako) and the peroxidase substrate diaminobenzidine (DAB, Dako), followed by counterstaining with hematoxylin and examination under a light microscope. A total of 400 nuclei from 5 fields of 5 sections of each mouse were counted in a blinded manner and the percentage of macrophage infiltrates was calculated.

### Flow cytometry analysis

Groups of C57BL/6 mice were fed with plain water or water containing 2 mg/ml of GABA for 4 weeks. Their splenic mononuclear cells were prepared and the frequency of CD4^+^Foxp3^+^ Tregs was characterized by flow cytometry analysis. Briefly, splenic mononuclear cells (10^6^/tube) were treated in duplicate with anti-CD16/32 on ice for 20 minutes. After washing, the cells were stained with FITC-anti-CD4 (BD PharMigen, San Diego, USA) for 25 minutes, fixed, permeabilized and stained with PE-anti-Foxp3 or isotype control (Biolegend, San Diego. USA), respectively. After washing, the cells were characterized by flow cytometry analysis and the percentage of CD4^+^Foxp3^+^ cells in total CD4^+^ T cells was calculated.

### Statistical analysis

Data are expressed as mean or mean ± SD of each group of mice. The difference between groups was analyzed by Students' t-test and X^2^ test. The potential correlation between measures was analyzed by Sperman's correlation analysis. A p value of less than 0.05 was considered statistically significant.

## Results

### Oral treatment with GABA does not change the amounts of water and food consumed, but significantly reduces the gain of body weight in HFD-fed mice

**Figure 1:**
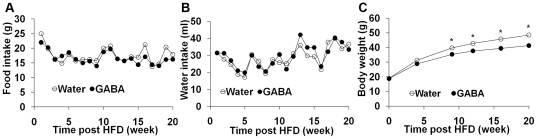
Oral treatment with GABA does not alter the amounts of water and food consumed, but reduces the gain of body weights in the HFD-fed mice. Groups of C57BL/6 mice were fed with HFD and provided with plain water or water containing 2 mg/ml of GABA for 20 weeks. The amounts of water and food consumed by individual mice and their body weights were measured longitudinally. Data are expressed as mean values of water and food consumed by individual mice per day and mean values of body weights of each group of mice (n = 20 per group) at the indicated time points post-HFD. Intragroup variations were less than 10% for the amounts of water and food consumption and less than 15% for the values of body weights. (A) The amounts of water consumed; (B) The amounts of food consumed; and (C) The body weights. *p< 0.05; ** p<0.01 vs. the GABA-fed mice.

GABA_A_-R subunits are expressed by immunocompetent cells, including T cells and macrophages and are also expressed by adipose tissues [13], [14], [15], [17]. Our previous studies and those of others have shown that treatment with GABA or a GABA_A_ receptor agonist inhibits inflammation in different mouse models of autoimmune diseases [16], [17], [18], [20], [21]. To determine whether GABA could modulate HFD-induced inflammation, obesity, and glucose intolerance, groups of 5-week-old male C57BL/6 mice were fed with HFD and provided with plain water or water containing 2 mg/ml of GABA for 20 weeks. Their food and water consumption was measured weekly (Fig. 1A and B). There was no significant difference in the mean amount of food intake and water consumption of individual mice between the water and GABA-treated groups of mice, consistent with our previous observations in a RA-prone stain of mice [18]. Although the body weights of individual mice in both groups of mice gradually increased following HFD-feeding, the mean body weights in the GABA-treated mice at 10 weeks post GABA treatment were significantly less than that of the water-fed mice (Fig. 1C) and remained significantly less than that of controls throughout the subsequent observation period. These data indicated that oral treatment with GABA did not significantly alter the food and water consumption, but reduced the HFD-associated gain in mouse body weight.

### Oral treatment with GABA improves glucose tolerance and insulin sensitivity in HFD-fed mice

**Figure 2:**
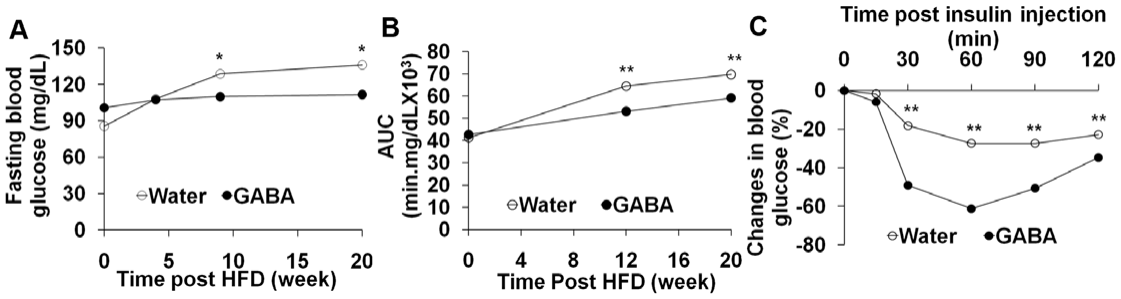
Oral treatment with GABA improves glucose tolerance and insulin sensitivity in HFD-fed mice. Groups of C57BL/6 mice were fed with HFD and given plain water or water containing 2 mg/ml of GABA for 20 weeks. The concentrations of fasting blood glucose, intraperitoneal glucose tolerance (IPGT), and insulin sensitivity of individual mice were tested longitudinally, as described in the Materials and Methods. Data are expressed as mean values of the concentrations of fasting blood glucose (A), the AUC of IPGT (B), and the percentages of blood glucose changes following insulin challenge (C) in different groups of mice (n = 20 for each group). *p< 0.05; ** p<0.01 vs. the GABA-fed mice.

During the study period, we measured the concentrations of fasting blood glucose, glucose tolerance and insulin sensitivity in the water-fed control and GABA-fed mice longitudinally. We found that the concentrations of fasting blood glucose gradually increased in control mice and some mice at 20 weeks post HFD developed T2DM. In contrast, the concentrations of fasting blood glucose in mice that received GABA remained at a similar level throughout the observation period (Fig. 2A). As a result, the concentrations of fasting blood glucose in the GABA-fed mice were significantly lower than that of controls at 9 weeks post HFD (p<0.05). Similarly, oral feeding with GABA inhibited the development of glucose intolerance in the HFD-fed mice (Fig. 2B). Furthermore, while challenge of the control mice with insulin only resulted in moderate reduction in the concentrations of blood glucose, an indication of insulin resistance, challenge with insulin dramatically reduced the concentrations of blood glucose in the GABA-fed mice. Together, these data clearly demonstrated that oral treatment with GABA inhibited the HFD-induced glucose intolerance and insulin resistance in mice.

### Treatment with GABA improves glucose tolerance and insulin sensitivity in mice after the onset of obesity and T2DM

**Figure 3:**
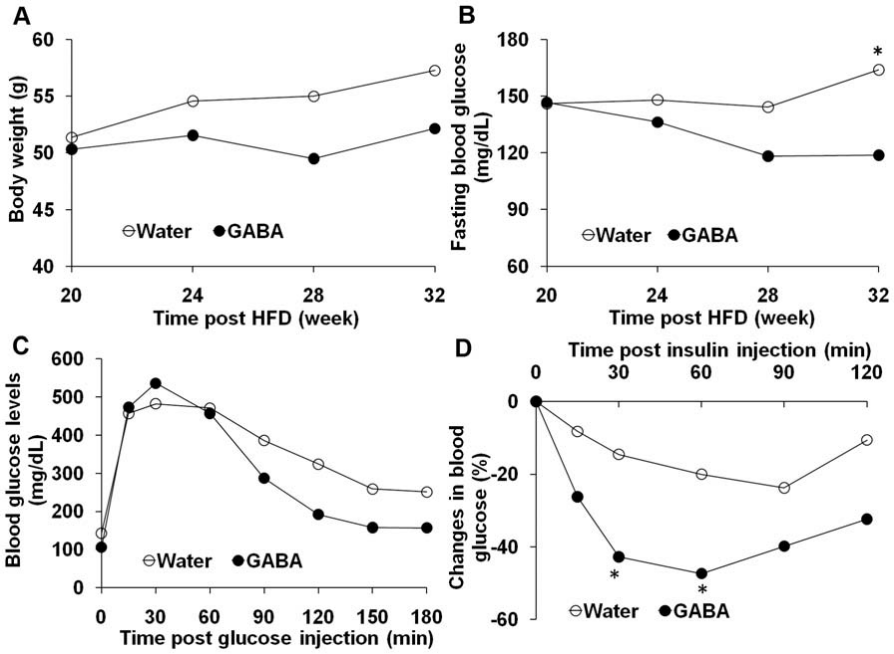
Oral treatment with GABA improves glucose tolerance and insulin sensitivity in mice after the onset of obesity and T2DM. C57BL/6 mice were fed with HFD for 20 weeks and their fasting blood glucose and IPTG were measured. Individual mice with body weight >48g, fasting glucose level of >145 mg/dL and abnormal IPTG (blood glucose >220 mg/dL at 2 hours post-IPGT) were considered to have obesity and T2DM, and randomly treated orally with plain water (control) or water containing 2 mg/ml of GABA for another 12 weeks. Their body weights, fasting glucose concentrations, IPGT and insulin sensitivity were measured longitudinally. (A) The body weights; (B) The concentrations of fasting blood glucose; (C) The dynamic changes following glucose challenge; and (D) The percentages of blood glucose concentrations following insulin challenge. Data are expressed as mean values of each group (n = 4 per group) at the indicated time points and the concentrations of blood glucose before insulin challenge in individual mice were used as 100%. *p< 0.05; ** p<0.01 vs. the GABA-fed mice.

To test whether treatment with GABA could modulate glucose tolerance and insulin sensitivity in mice after the onset of obesity and T2DM, C57BL/6 mice were fed with HFD for 20 weeks and their fasting glucose, glucose tolerance, and insulin sensitivity were measured. Individual mice with obesity (body weight > 48 g) and T2DM (abnormal fasting blood glucose and impaired glucose tolerance and insulin sensitivity, see Materials and Methods) were randomized and fed with water as controls or with water containing GABA (2 mg/ml) for another 12 weeks. Their body weights, fasting blood glucose levels, glucose tolerance, and insulin sensitivity were measured longitudinally. As shown in Fig. 3, mice that had been continually fed with HFD and plain water gradually increased their body weights while the mice fed with HFD and water containing GABA gained little or no body weight (Fig. 3A). More importantly, following treatment with GABA, all of diabetic mice became euglycemic and the mean concentrations of fasting blood glucose in the GABA-treated mice were significantly lower than that of controls at 32 weeks post HFD (p<0.05, Fig. 3B). In addition, treatment with GABA improved glucose intolerance and insulin sensitivity in the HFD-fed mice (Fig. 3C and D). While both groups of mice displayed similar levels of peak blood glucose after glucose challenge, the GABA-treated mice exhibited near normal levels of blood glucose at 2 hour post glucose challenge. In contrast, the concentrations of blood glucose at 2-hour post glucose challenge in the plain water-fed mice remained hyperglycemic. Moreover, while insulin challenge reduced the concentrations of blood glucose by less than 25% in the plain water-fed mice the same treatment dramatically reduced the concentrations of blood glucose by nearly 50% during the early stages of responding to insulin in the GABA-treated mice. Therefore, treatment with GABA improved glucose intolerance and insulin sensitivity in the mice with established obesity and T2DM.

### Treatment with GABA reduces adipocyte mass and macrophage infiltration in the adipose tissues of HFD-fed mice

**Figure 4:**
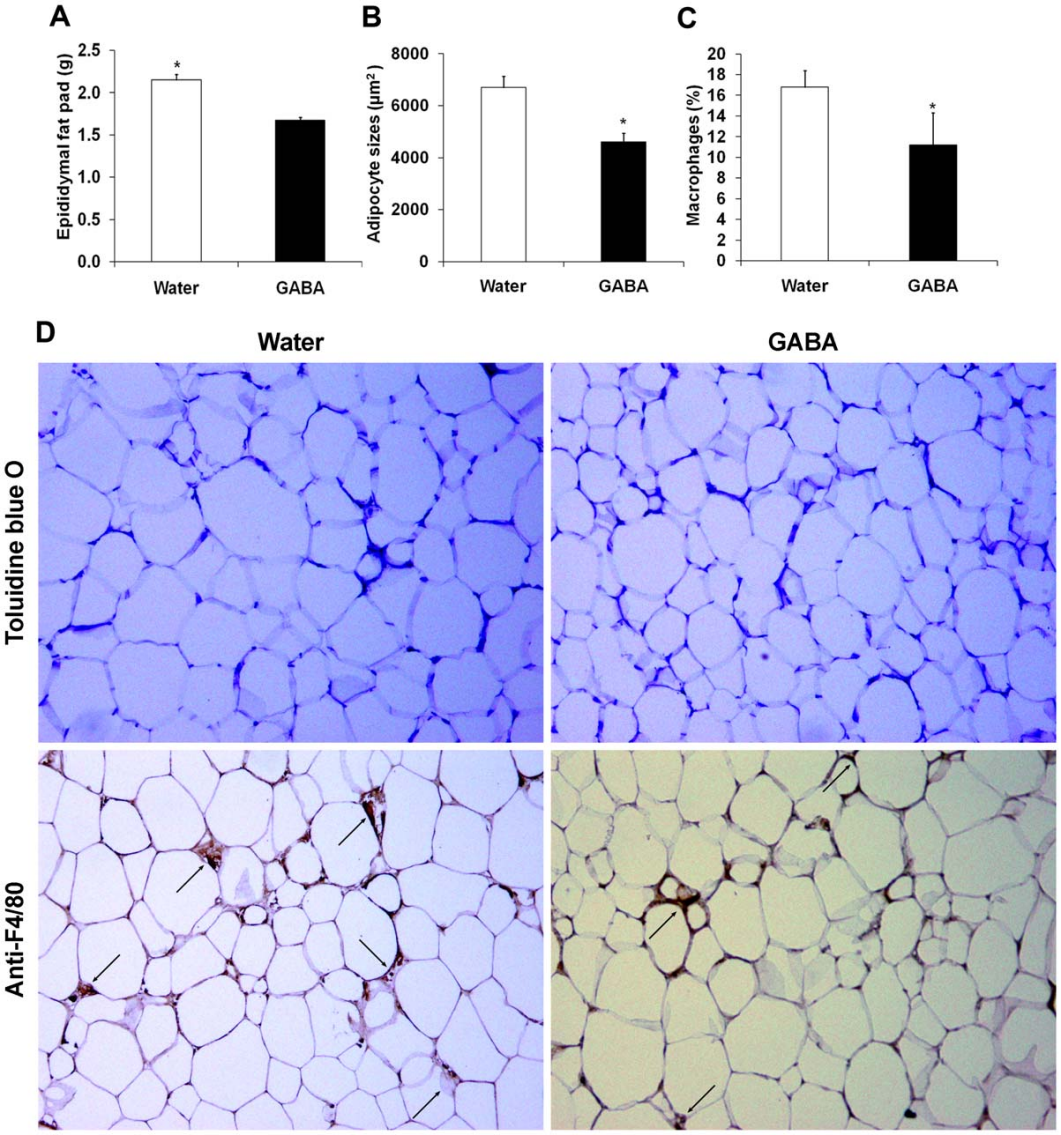
Oral treatment with GABA reduces adipocyte mass and macrophage infiltration in the adipose tissues of HFD-fed mice. Groups of C57BL/6 mice were fed with HFD and provided with plain water or water containing 2 mg/ml of GABA for 20 weeks. The mice were sacrificed and the amounts of VAT in individual mice were measured. One portion of the VAT was fixed with 4% paraformaldehyde for 24 hours and the VAT sections at 5 µm were stained with Toluidine blue O, followed by examination under a light microscope. The sizes of adipocytes in 5 sections of individual mice from each group (n = 8) were examined in a blinded fashion. Another portion of VAT was fixed with the Bouin buffer for 48 hours and the VAT sections were subjected to immunohistochemistry analysis of infiltrated macrophages using anti-F4/80 antibodies and DAB substrate (brown), and the percentages of macrophages in 400 nuclear cells from 5 sections of each mouse in individual groups of mice were quantified in a blinded manner. Data are representative images of the adipocytes, macrophages stained and expressed as mean ± SEM from each group of mice (n = 8 for immunohistological examination and n = 12 for measuring the amounts of VAT per group). *p< 0.05; ** p<0.01 vs. the GABA-fed mice.

Previous studies have shown that macrophage-related chronic inflammation in adipose tissues is crucial for the development of insulin resistance, obesity, and T2DM [1], [22]. Given that GABA_A_-R subunits are expressed by macrophages and adipose tissues [13], [15], we further tested whether GABA could modulate macrophage-associated inflammation in adipose tissues, or alter adipose tissue mass in the HFD-fed mice. Following HFD feeding for 20 weeks, control and GABA-treated mice were sacrificed and their VAT was dissected out and weighed. The total amount of VAT in the GABA-treated mice was significantly less than that of the controls (Fig. 4A), consistent with reduced body weights. Further analysis of VAT revealed that the size of adipocytes in the GABA-treated mice was also significantly less than that of the controls (Fig. 4B). In addition, characterization of F4/80^+^ macrophages in the adipose tissues indicated that the percentage of macrophages infiltrated in the adipose tissues of the GABA-treated mice was significantly less than that of the controls (Fig. 4C). Collectively, these data clearly indicated that treatment with GABA reduced the adipose tissue mass and macrophage infiltrates in the adipose tissues of HFD-fed mice.

### Oral treatment with GABA increases the frequency of splenic Tregs in vivo

**Figure 5:**
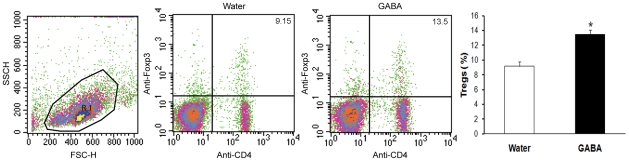
Oral treatment with GABA increases the frequency of splenic Tregs *in vivo*. C57BL/6 mice were fed with plain water or water containing GABA (2mg/ml) for four weeks. Their splenic mononuclear cells were prepared and treated with anti-CD16/32. Subsequently, splenic mononuclear cells (10^6^/tube) were stained in duplicate with FITC-anti-CD4 and after washing, the cells were fixed, permeabilized and stained with PE-anti-Foxp3, followed by flow cytometry analysis. The cells were stained with single fluorescent-labeled antibodies or with isotype-matched IgG as controls. Data are representative charts and expressed as mean ± SEM of the percentage of CD4^+^Foxp3^+^ Tregs in splenic CD4^+^ T cells of different groups of mice (n = 3–4 mice per group) from two separate experiments. *p<0.01 vs. the water-fed mice.

The Foxp3^+^ Tregs are negative regulators of inflammatory responses and recent studies have indicated that Tregs can inhibit obesity-related inflammation and insulin resistance in mice [12], [23]. To further understand the mechanisms underlying the actions of GABA in regulating the HFD-induced obesity and insulin resistance, groups of C57BL/6 mice were fed with plain water or water containing GABA (2 mg/ml) for 4 weeks and their splenic CD4^+^Foxp3^+^ Tregs were characterized by flow cytometry analysis. As shown in Fig. 5, there was no significant difference in the frequency of CD4^+^ T cells between the plain water-fed controls and GABA-treated mice. However, the frequency of CD4^+^Foxp3^+^ Tregs in the GABA-treated mice was significantly higher than that of the controls (p<0.01). Therefore, treatment with GABA increased the frequency of Tregs in mice.

## Discussion

Macrophage-related chronic inflammation in adipose tissues is associated with the development of insulin resistance and glucose intolerance, leading to the development of obesity and T2DM. Our previous studies, and those of others, have shown that GABA, through the activation of GABA_A_-R, inhibits inflammation in mouse models of autoimmune diseases [16], [17], [18], [19], [20]. In this study, we examined the impact of oral treatment with GABA on the development of HFD-induced glucose intolerance and insulin resistance as well as obesity in mice. We found that oral treatment with GABA did not modulate the amount of food intake and water consumption by mice, consistent with our previous findings [18]. Given that GABA has little or no ability to pass through the blood brain barrier, it is not surprising that activation of peripheral GABA receptors did not alter calorie intake in mice.

Furthermore, while most of the HFD-fed control mice displayed a significant increase in body weights, the mice that had been fed with HFD and GABA had significantly less gain in body weights over the observation period. Over-consumption of calories can cause obesity and lead to the development of insulin resistance and T2DM [22], [24]. We found that oral treatment with GABA significantly improved glucose intolerance and insulin resistance in HFD-fed mice. Evidentially, the levels of fasting blood glucose, blood glucose at 2 hours post-glucose challenge, and the relative levels of blood glucose post-insulin treatment in the GABA-treated mice were significantly lower than that of the control mice. More importantly, after the establishment of obesity and T2DM, oral treatment with GABA significantly reduced the gain in body weight and improved glucose tolerance and insulin sensitivity in mice. These novel data demonstrated that oral treatment with GABA not only prevented the HFD-induced obesity and T2DM development, but also inhibited the progression of obesity and T2DM in mice. Given that GABA is safe for human consumption, GABA may be valuable for the prevention and treatment of obesity and T2DM in the clinic.

Recent studies have shown that adipose tissue-related chronic inflammation contributes to the development of insulin resistance, a key component of metabolic syndrome, and leads to the development of obesity and T2DM as well as other metabolic disorder-related diseases [1], [22]. To understand the potential mechanisms underlying the therapeutic effect of GABA in inhibiting the HFD-induced obesity and T2DM, we examined the mass of adipose tissues and inflammatory infiltrates. In comparison with that in the control mice, oral treatment with GABA significantly reduced the total amounts of epididymal fat tissues and the size of adipocytes in the HFD-fed mice. Because macrophages are predominant players in the development of chronic inflammation in adipose tissues [10], [11], [25], [26], [27], we further characterized the frequency of macrophage infiltrates in the epididymal fat tissues. We found that oral treatment with GABA significantly decreased the frequency of macrophage infiltrates in adipose tissues of the HFD-fed mice. More importantly, GABA_A_ receptor subunits are expressed by adipocytes and macrophages, and GABA_A_ receptor agonists can inhibit macrophage activation [13], [15], [17]. It is possible that GABA, through the GABA_A_ receptors, inhibits macrophage activation and migration, reducing chronic inflammation in adipose tissues. Given that inflammatory adipocytes can produce adipokines that recruit inflammatory infiltrates, such as macrophages, GABA may alternatively inhibit adipogenesis and adipokine production, indirectly limiting the macrophage infiltration into adipose tissue in the HFD-fed mice. Recent studies have highlighted the importance of glucagon in regulating glucose homeostasis [28], [29]. Physiologically, GABA acts synergistically with insulin to inhibit the secretion of glucagon by the pancreatic α-cells, and reverse insulin-deficiency-related hyperglycemia in mice [29], [30]. Although we can not completely exclude the possible effect of GABA-mediated inhibition of glucagon secretion, our data from islet transplant suggest that treatment with GABA alone has little ability to reverse T1D in diabetic NOD mice [19]. Hence, the effect of GABA-mediated inhibition of glucagon secretion may be minor in inhibiting HFD-induced glucose intolerance, insulin resistance, and macrophage-related inflammation. Conceivably, GABA, through its receptors, on macrophages and adipocytes, improves glucose tolerance and insulin sensitivity in our experimental model. We are interested in further investigating how activation of GABA_A_ receptors modulates adipogenesis and macrophage activation and migration as well as adipokine and cytokine production by adipocytes.

Notably, Tregs are potent inhibitors for macrophage activation and function [23]. Tregs are negative regulators of obesity-related insulin resistance and T2DM in mice [12], [31]. We found that oral treatment with GABA significantly increased the frequency of splenic Tregs in C57BL/6 mice, indicating that GABA promoted Treg proliferation and maturation *in vivo*. Our previous study has shown that GABA, through the GABA_A_ receptors, induces effector T cell cycle arrest, consistent with the notion that engagement of GABA_A_ receptors induces the hyperpolarization of membrane potentials, which may inhibit the TCR-related signaling [16]. The significantly increased frequency of splenic Tregs by GABA treatment suggests that GABA, through the GABA_A_ receptors, may promote the depolarization of membrane potentials by opening the voltage-dependent calcium channel and enhancing the TCR-triggering calcium-dependent downstream survival and growth signaling [16], [32]. Given that Tregs are potent inhibitors of inflammation and insulin resistance the GABA-induced increase in Tregs may also inhibit macrophage infiltration and related chronic inflammation, contributing to the therapeutic effect of GABA in inhibiting the HFD-induced obesity and T2DM.

In summary, our data demonstrated that oral treatment with GABA inhibited the HFD-induced obesity and improved glucose intolerance and insulin sensitivity, even after the establishment of obesity and T2DM in mice. Furthermore, oral treatment with GABA reduced the HFD-induced adipocyte hypertrophy and adipose tissue mass, accompanied by significantly reduced macrophage infiltrates in the adipose tissues. Furthermore, we found that GABA treatment increased the frequency of splenic Tregs in mice. Apparently, GABA, through its GABA_A_-Rs on adipocytes, macrophages and T cells, inhibited chronic inflammation in adipose tissues, leading to the improvement of glucose tolerance and insulin sensitivity in HFD-fed mice. Given that GABA mainly acts on the peripheral GABA receptors and is safe for human consumption, GABA and other GABA_A_-R agonists may be valuable for the prevention and treatment of obesity and T2DM in the clinic.
